# Design rules applied to silver nanoparticles synthesis: A practical example of machine learning application.

**DOI:** 10.1016/j.csbj.2024.02.010

**Published:** 2024-02-17

**Authors:** Irini Furxhi, Lara Faccani, Ilaria Zanoni, Andrea Brigliadori, Maurizio Vespignani, Anna Luisa Costa

**Affiliations:** a CNR-ISSMC (Former ISTEC), National Research Council of Italy-Institute of Science, Technology and Sustainability for Ceramics, Faenza, Italy; b Transgero Limited, Limerick, Ireland

**Keywords:** Silver nanoparticles, synthesis, machine learning, Shapley values, Safe and sustainable

## Abstract

The synthesis of silver nanoparticles with controlled physicochemical properties is essential for governing their intended functionalities and safety profiles. However, synthesis process involves multiple parameters that could influence the resulting properties. This challenge could be addressed with the development of predictive models that forecast endpoints based on key synthesis parameters. In this study, we manually extracted synthesis-related data from the literature and leveraged various machine learning algorithms. Data extraction included parameters such as reactant concentrations, experimental conditions, as well as physicochemical properties. The antibacterial efficiencies and toxicological profiles of the synthesized nanoparticles were also extracted. In a second step, based on data completeness, we employed regression algorithms to establish relationships between synthesis parameters and desired endpoints and to build predictive models. The models for core size and antibacterial efficiency were trained and validated using a cross-validation approach. Finally, the features’ impact was evaluated via Shapley values to provide insights into the contribution of features to the predictions. Factors such as synthesis duration, scale of synthesis and the choice of capping agents emerged as the most significant predictors. This study demonstrated the potential of machine learning to aid in the rational design of synthesis process and paves the way for the safe-by-design principles development by providing insights into the optimization of the synthesis process to achieve the desired properties. Finally, this study provides a valuable dataset compiled from literature sources with significant time and effort from multiple researchers. Access to such datasets notably aids computational advances in the field of nanotechnology.

## Graphical Abstract

**Figure:**
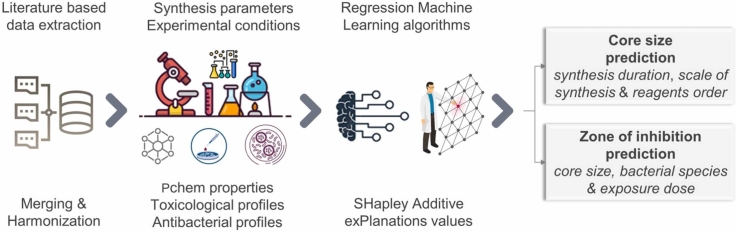

## 1 Introduction

Already available on the market, silver nanoparticles (AgNPs) embedded into products offer a variety of benefits such as an antimicrobial functionalities by eradicating or inhibiting the growth of bacteria [1], fungi [2], and viruses [3], as well as the ability to prolong the shelf life of products (i.e., food packaging). Their versatility renders them valuable in various products’ applications including wound dressings [4], medical devices [5], [6], water purification systems [7], and antibacterial coatings [8]. AgNPs are also utilised as drug delivery carriers due to their size which enable them to readily penetrate cells and deliver therapeutic substances to targeted sites, enhancing treatments’ efficacy [9]. Additionally, because their unique optical and electrical properties, they are utilized in sensing and detection systems [10], measuring small quantities of target molecules, including heavy metals [11], environmental pollutants and biomarkers [12]. This has implications in fields like environmental monitoring, food safety and medical diagnostics [13]. They are also helpful in applications such as imaging devices [14] and solar cells [15]. Finally, have the ability to function as catalysts in a variety of industrial processes, such as organic synthesis, pollution control, and chemical manufacturing [16], [17], [18]. AgNPs have attracted attention as evidenced by the significant demand for and investment in associated research. The demand for AgNPs has been gradually increasing over the past 15 years, and manufacturing of the product is expected to exceed 500 tons annually to meet the demands of various industries [19]. Due to the growth of the market worldwide and the current offer of products with incorporated AgNPs, the study of their biological activity, functionalities and safety has become a paramount matter of research [20]. However, their activities are modulated by their physicochemical properties (pchem) which are defined in the initial life cycle stage, the synthesis. To exploit and fulfil the Safe by Design (SbD) concept at the earliest stages of innovation it is imperative to comprehend which pchem properties, which are indirectly defined by key synthesis parameters (temperature, precursors, concentrations, treatments, etc), impact the toxicological and/or antibacterial profile. This challenge can be addressed with the development of predictive models that forecast desired endpoints while capturing the relationship among pchem and key synthesis parameters. From the standpoint of computational-based drug development approach, it is crucial to create predictive models that can screen a variety of potential experimental conditions and help identify the selection of optimal ones to employ in the laboratory experiments. In order to create such models, three key parts with their related data have to be investigated, i) synthesis methods of AgNPs along with experimental conditions, ii) resulting measured pchem properties of the synthesised AgNPs and iii) their functional or safety profiles.

### 1.1 Synthesis methods & experimental conditions

Top-down and bottom-up methods by either chemical, physical or biological means are the main synthesis approaches for AgNPs. A top-down example includes the grinding of bulk metals and the subsequent inclusion of colloidal protective agents to stabilize the resulting nanosized metal particles. The bottom-up approach includes reduction of metal precursors, electrochemical or green synthesis methods [21], [22] etc., with the main ones being: i. chemical reduction of a silver salt such as silver nitrate (AgNO_3_) using a reducing agent (sodium borohydride (NaBH_4_) or sodium citrate (Na_3_C_6_H_5_O_7_)) [23]. ii. sol-gel technique, which combines a gel-forming agent solution with a silver precursor (i.e., silica precursor). After that, the mixture is exposed to condensation and hydrolysis, which forms a gel containing silver ions. To convert ions into AgNPs, the gel is subjected to further heat treatment or reduction [24]. iii) green synthesis, which involves the use of natural or biological materials as reducing agents or stabilizers (i.e., plant extracts) [25]. iv) electrochemical deposition which involves the reduction of silver ions present in an electrolyte solution onto an electrode surface, typically a cathode [26]. During the synthesis process, several parameters can be adjusted to control and fine-tune the resulting pchem properties of AgNPs, such as:

1) the reagents’ concentration which includes the silver precursor or the reducing agents. These chemical agents have an impact on the size, shape and dispersity index of the resulting NPs; higher concentrations frequently resulting in smaller NPs [27].
2) the pH of the reaction solution. The stability, aggregation behaviour, and interaction of the particles with other substances can all be affected by changes in the surface charge of the particles caused by the pH of the solution [28].
3) the reaction temperature. Temperature plays a crucial role in regulating the growth and nucleation kinetics; Higher temperatures often result in faster nucleation and growth, leading to larger NPs [29].
4) the usage of stabilizing agents (i.e. polymers or surfactants). Those agents can control stability and prevent aggregation, through their effects on steric hindrance and/or electrostatic repulsion, which can also affect surface chemistry and dispersibility [30].
5) the reaction time. Time has a strong impact on the pchem properties with longer reaction times often resulting to larger particles due to continued growth and reprecipitation processes associated with the Ostwald ripening [31], [32].
6) the use of external fields such as electric, magnetic or ultrasound. AgNPs with desired size and morphology can be produced by adjusting the applied voltage and deposition duration [33], [34].
7) the use of natural or biological materials as reducing agents or stabilizers in green synthesis methods can introduce unique properties due to synergistic interactions [35].

Based on the information summarized above, it is can be hypothesized that various experimental conditions can be adjusted to alter the size, shape, and other o pchem properties of synthesised NPs.

### 1.2 Physicochemical properties

One of the main pchem properties affecting NPs' behaviour is their core size. AgNPs are typically employed in products’ applications ranging from 1 to 10 nm in size [36]. Numerous studies have revealed that smaller particles exhibit greater antibacterial activity but higher cytotoxic responses [37], [38]. As the particle size gets smaller the specific surface area increases, hence, a greater proportion of its atoms are displayed on the surface [39]. This implies that biological interactions and toxicity are dependent on the particles’ surface area than on the particle mass. The dispersibility, solubility, and/or hydrophobicity can be altered by the AgNPs’ functionalization with various coatings. The coatings in turn, affect AgNPs’ bioaccessibility and biodurability. There is a considerable literature amount indicates that the type of coating impacts both fate and toxicity of AgNPs [40], [41], [42]. For instance, trisodium citrate (CT-AgNP) and polyvinylpyrrolidone (PVP-AgNP) improve biocompatibility and stability against agglomeration phenomenon [43]. Moreover, coating modifies the surface charge which influences their cytotoxic potential and biological targets; positively charged NPs have stronger affinity for negative bacterial membranes causing higher cellular contents leakage and bacterial death [44]. Studies employing AgNPs of different sizes also revealed shape-dependent effects. For example, compared to spheres and wires, 55 nm silver nanotubes had a stronger antibacterial activity against *Escherichia coli* [45]. On the other hand, spherical, triangular and cuboid AgNPs were found to have no biocidal effect on *Staphylococcus aureus* [46]. The agglomeration state of NPs also determines cellular uptake as well as biological reactions [47]. A number of studies have shown that agglomerated particles exhibit reduced cytotoxicity compared to free AgNPs [47], [48]. In summary, knowledge of the pchem properties is essential to comprehending the biological reactions of AgNPs and the underlying mechanisms of action which determined their applications.

### 1.3 AgNPs mechanisms of action and applications

AgNPs stand out among the many NMs that are known to be useful in preventing the growth of several bacteria due to their potent inhibitory and bactericidal properties [49], [50]. AgNPs are also familiar for their antifungal and antiviral activities [51]. Several studies have reported their potent antifungal activity against several phytopathogenic fungi (e.g. *Alternaria alternate*, *Sclerotinia sclerotiorum*, *etc*) as well as human pathogenic fungi (e.g. *Candida* and *Trichoderma sp.*) [52] and against several viruses including SARS-CoV-2 [53], [54]. Research has indicated that AgNPs interact with bacterial membranes but the precise mechanism underlying these activities remains unclear [55], [56]. One leading hypothesis holds that AgNPs when in contact with bacteria, produce free radicals and reactive oxygen species, which damage the bacteria's internal organelles and change the intracellular signalling pathways that trigger apoptosis [57]. Another mechanism is the adhesion of AgNPs to the bacterial wall, followed by the infiltration of particles that act as a Trojan horse and release ions, causing bacterial membrane damage and leakage of cellular contents and death [58]. For example, the antibacterial activity of 12 nm AgNPs was excellent against both Gram-positive and Gram-negative bacteria including *Staphylococcus bacillus*, *Staphylococcus aureus*, and *Pseudomonas aeruginosa* [59]. This indicates that both membrane thickness and surface charge facilitate particle attachment onto the membrane [60]. Finally, the release of Ag^+^ ions from the surface area is another crucial factor that contributes to the antibacterial activity. Release rate is dependent on a number of factors including size, shape, capping agent and colloidal state of NPs [51], [61]. It is well established, smaller AgNPs have faster Ag^+^ release rate and, hence, an increased bioavailability, and also cytotoxicity, compared with larger NPs [62], [63].

It becomes evident from the above sections that a modelling approach is essential to effectively and systematically capture the transversal information involved in the synthesis stage and to consider the impact of the many experimental parameters involved in the synthesis process on the pchem properties of the resulting AgNPs, their antibacterial efficiencies, and/or their toxicity effects.

### 1.4 Machine learning models

With conventional approaches it is challenging to systematically explore the entire parameter space and comprehend the relationships between experimental synthesis conditions and resulting AgNPs characteristics due to the complexity and multidimensionality. Additionally, manual experimentation can be time-consuming, costly, and limited in scope, making it difficult to optimize synthesis conditions effectively. Hence, modelling techniques, such as machine learning (ML) models provide a promising means to navigate this complexity and capture the interplay of multiple parameters.

ML models can be trained using existing data on synthesis experimental parameters and corresponding pchem properties or functionalities, allowing the development of predictive models that can guide and optimize the synthesis process [64], [65]. The models can then be used to predict the AgNPs’ properties synthesized under different conditions. For example, by inputting the reagent concentrations, reaction temperatures, reaction duration etc., into a model, it can predict the resulting size, shape, or stability [66], [67]. ML can be used to optimize the synthesis process by iteratively adjusting and identifying the optimal combination of synthesis parameters to converge into the desired properties. Designing effective experiments (Design of Experiments), to investigate the synthesis parameter space, can be achieved by ML which recommends the most instructive experiments to be conducted based on the knowledge gained from the available data. Such as approach minimizes the number of experiments required and speeds up the process of discovering the combination of synthesis conditions. Nanoparticles have only partially captured the benefits of automation, as exhaustively discussed in a review on the role of ML in Nanosafety [68]. One notable example of ML application is a proposed framework that optimizes the synthesis and extracts knowledge of the relationship between composition and optical properties [69]. The neural networks’ output layer is composed of 421 nodes, which are corresponding to the UV–Vis spectral data points. The experimental input parameters contained variables of different flow rates such as silver nitrate, trisodium citrate etc., The framework was built based on flow rates as input features and specific experimentation apparatus to obtain synthesized in aqueous sub-microliter droplets NPs. In an attempt to increase the application domain of ML in the synthesis process, we manually collected synthesis data from multiple literature studies and leveraged various ML algorithms to explore the parameter space of synthesis conditions during the experiments and the resulting AgNPs characteristics followed by a targeted functionality (such as antibacterial properties). Data extraction included parameters such as reagent concentrations, reaction conditions, typology of stabilizing agents, etc. Additionally, pchem properties such as the core size measurements obtained from various characterization techniques, such as transmission electron microscopy (TEM), scanning electron microscopy (SEM), were recorded. Antibacterial efficiency and toxicological profiles were also collected when available in the study design. In a second step, based on data completeness, we employed regression ML algorithms to build predictive models targeting various endpoints to establish relationships between synthesis parameters and desired outputs. The developed models were validated using a cross-validation approach. Finally, the feature’s impact, based on SHapley Additive exPlanations (SHAP) average values, was utilized to provide insights into the contribution of individual features in the model’s predictions.

## 2 Materials and methods

### 2.1 Literature review

A comprehensive literature review was performed for the duration of 2004–2022 using a combination of keywords such as “silver nanoparticles”, “silver nanomaterials”, “synthesis”, “nanoparticle synthesis”, “nanoparticle”, “chemical reduction”, and “green synthesis” to identify relevant studies and extract key information. The search was performed across various academic databases, including PubMed, Scopus and Google Scholar. The aim was to compile a variety of synthesis protocols including the experimental conditions and the steps followed, the characterization techniques employed for pchem properties identification and to record functionalities or toxicological properties of the aforementioned synthesized NPs. The retrieved articles were screened based on their titles and abstracts to assess their relevance to AgNPs synthesis. Selected articles were then obtained in full text and further reviewed to determine their suitability for inclusion in the literature review. Additionally, the reference lists of these articles were examined to identify additional relevant studies that may have been missed in the initial search. The inclusion criteria encompassed a variety of publication types, including research articles and review papers, excluding book chapters and conference proceedings.

### 2.2 Data extraction – first dataset

A manual data extraction was performed systematically from the selected articles. Relevant information, including synthesis parameters such as experimental conditions, AgNPs pchem properties, characterization techniques, toxicological outcomes, and antibacterial efficiency measurements were recorded in an Excel workbook format. To ensure consistency and reduce potential biases from expert judgment, the literature review was conducted by a team of researchers with expertise in nanomaterial synthesis and characterization. Regular discussions were held to address any discrepancies or uncertainties during the data extraction process. Having data on the synthesis procedure, the first step of the innovation process, is crucial for developing computational models for predicting multiple endpoints such as resulting pchem properties or functionalities. The data extraction included synthesis parameters such as:

• Reagent concentrations and the chemical compounds. As reagents, we considered the silver precursor, the reducing agents used, the capping/stabilizing agent, and any possible additional catalyst during the reaction process.
• Reaction conditions included temperature, any external source of energy applied (ultrasound, pressure etc.,), the pH of the reaction, reaction duration, stirring approach and stirring conditions, and any specific environmental conditions (e.g., light conditions).
• Post-synthesis treatments were recorded such as drying or washing.
• Resulting pchem properties of the synthesized AgNPs were recorded. The characterization techniques employed to analyse the synthesized nanoparticles were specified such as transmission electron microscopy (TEM), scanning electron microscopy (SEM), X-ray diffraction (XRD), dynamic light scattering (DLS), etc., providing information about AgNPs size, shape, zeta potential etc.
• Finally, antibacterial and toxicological results were also extracted, reporting all the experimental details such as the cell line or organisms exposed, the exposure conditions (dose and time) and the endpoints measured along with the assays such as cell viability, inflammation indication (in case of toxicological studies) or the antibacterial efficiency or zone of inhibition (in case of antibacterial assessments).

### 2.3 Data harmonisation and exploration

Following data extraction, the features were then harmonized to create a unified and cohesive dataset, to ensure compatibility and consistency, and to allow the exploration of features and the development of ML models. Experimental data collected from different studies exhibit variations in terms of synthesis parameters, characterization techniques, units of measurement, and reported properties. During meetings and discussions, the team collaborated to harmonize the extracted data and to reach a consensus. It was crucial to ensure that the dataset covered a broad range of synthesis conditions while maintaining data integrity. To achieve harmonization, the following main steps were followed as a principle:

• converting various units of measurements into a consistent format (SI unit). For example, ensuring that reagent concentrations are expressed in the same units (e.g., molar concentration) or that temperature is consistently reported in a specific scale (e.g., Celsius or Kelvin). This step minimized discrepancies arising from different reporting conventions.
• aligning the different terminologies used to express the same ontology. For example, synthesis parameters, such as reagent names, reducing agents, stabilizing agents, and experimental conditions, needed to be harmonized to ensure consistency across the dataset. This involved creating a standardized vocabulary to facilitate data integration.
• integration of features into simpler categories to avoid high cardinality features for the training of the models, for example, the capping agents might greatly vary across the studies for this reason, agents were simplified, classified, and aggregated into organic and inorganic classes (more information in the results Section 3.2).

Finally, all the data were inserted in a final workbook (*Raw data* tab in the supplementary material.

### 2.4 Machine learning exploration

The final workbook was split into i) synthesis dataset, ii) toxicological dataset, and iii) antibacterial dataset to explore the potential of developing and applying modelling tools based on data completeness. Data completeness in this work refers solely to the extent to which data required for modelling is present within the dataset with low data completeness suggesting missing information that hinders the application of modelling. In the nanosafety field data completeness can also refer to minimum reporting standards or guidelines for specific experiments or applications (e.g. OECD, ISO, REACH, MIRIBEL). When data sufficient for modelling exploration the selected dataset was then pre-processed via one-hot encoding for the categorical variables enabling the inclusion of these in predictive regression modelling tasks. One-hot encoding is a commonly used technique for handling categorical values converting them into a numeric label. In a second step, PyCaret regression library [70] was utilized to train various ML algorithms. PyCaret contains more than 18 regression models such as linear regression, decision tree, random forest, support vector, extra trees, ridge, elastic net, huber, bayesian ridge among others. These algorithms cover a wide range of popular regression techniques, enabling the comparison of different models. The models were trained with the training set and validated via 10-fold cross validation approach. In this method, the dataset is divided into ten subsets of equal size. The model is then trained on nine of these subsets (training set) and evaluated on the remaining subset (test set). This process is repeated ten times, with each subset serving as the validation test set once. By averaging the performance metrics across these iterations, such as accuracy or mean squared error, the cross-validation approach provides an estimation of the model's performance on unseen data. This estimation accounts for variability in the data and helps assess the generalization ability and predictability of the model. Validation metrics such as root mean squared error (RMSE), mean absolute error (MAE), and R-squared (R^2^), were used to assess and compare the models' accuracy. In a final step, the features’ impact based on SHAP (SHapley Additive exPlanations) average values [71], [72] was derived to provide insights into the overall contribution of individual features in the model's predictions. A positive average impact value suggests that the feature leads to more accurate predictions to the model's output, while a negative value indicates the opposite. Features with higher average impact values have a stronger influence on the model's predictions, indicating their significance in determining the target variable. The feature SHAP average impact values help identify key drivers or variables that significantly contribute to the model's output and gain insights into the underlying relationships between the features and the output. The code for the ML pipeline containing the preprocessing steps and models exploration can be found here.^2^

## 3 Results

### 3.1 Literature review & data extraction

From the literature review, 219 studies were collected from the 2004–2022 period. In the first workbook, both synthesis and potential outcomes, covering antibacterial and toxicological assessments, were extracted. The process of including data into the workbook file involved duplicating each row based on a specific parameter, while keeping the remaining columns unchanged. For example, if the same synthesis process and resulting pchem properties were exposed into a cell culture under different exposure doses (i.e., 10, 50, 100 mg/ML), this resulted in three rows with only exposure dose being changed (same process for the synthesis methodology or the antimicrobial studies). This technique allowed for the systematic generation of multiple rows, each representing a unique combination of the duplicated parameter and the constant values of the other columns. By expanding rows in this manner, the dataset encompass various scenarios and conditions related to the parameter of interest, while maintaining consistency in the other attributes. This approach enables comprehensive analysis and facilitates further exploration of the data by considering the impact of different values of the duplicated parameter on the corresponding outcomes or observations.

Below, in Table 1 a brief overview of the targeted information extracted:

**Table 1:** Overall overview of the first workbook design created by an iterative discussion with the research group.

Synthesis studies			Physicochemical properties characterization		
**The synthesis method**	Synthesis process	Chemical reduction, sol-gel, green synthesis, phytochemical or others	**Pchem properties**	Chemical composition	The chemical composition followed by the method of determination and instrumentation used
	Steps involved	Single-step or multiple steps process		Dissolution	Concentration of silver cations dissolved in relevant media (μM)
	Synthesis duration	The duration of the entire synthesis process (h)		Shape	Shape followed by method of determination and instrumentation used
	Scale	The scale of the synthesis process (ML)		Crystal structure	Crystal structure followed by method of determination and instrumentation used
**Precursor**	Precursor	The precursor compound utilised (usually AgNO_3_)		Zeta potential	Zeta potential followed by method of determination and instrumentation used. In addition, standard deviation, pH and isoelectric point are reported (mV)
	Source	The precursor commercial source (Sigma Aldrich, Aladdin, ACE chemicals, etc)		Core size	Core size followed by standard deviation, method of determination and instrumentation used (nm)
	Purity	The precursor purity ( %)		Crystallite size	Crystallite size followed by standard deviation, method of determination and instrumentation used (nm)
	Concentration	The precursor concentration along with its units of measurement (mM, where not explicit or calculable we express the amount of precursor in g)		Hydrodynamic diameter	Hydrodynamic diameter followed by standard deviation, method of determination and instrumentation used (nm)
**Capping agent**	Compound	The capping agent used during synthesis		Surface area	Surface area followed by standard deviation, method of determination and instrumentation used (m^2^/g)
	Classification	Capping agent classification into plant-based, chemical, fungal extract, bacterial-based, etc		Polydispersity index	Polydispersity index followed by standard deviation, method of determination and instrumentation used
	Class	Organic versus inorganic agents		Agglomeration	Information if agglomeration was present
	Concentration	The capping agent concentration along with its units of measurement (mM or mg/ML)		UV-Vis peaks	The peaks of UV-Vis analysis for NPs and of reducing agents (nm)
**Reducing agent**	Compound	The reducing agent used during synthesis			
	Classification	Reducing agent classification into plant-based, chemical, fungal extract, bacterial-based, etc	**Antimicrobial studies followed**		
	Class	Organic versus inorganic agents	**Exposure conditions**	Exposure dose	The exposure concentration along with its units of measurement (mg/ML)
	Concentration	The reducing agent concentration along with its units of measurement (ML or mg/ML)		Exposure duration	The exposure duration of the experiment (h)
**Catalyst**	Compound	The catalyst used during synthesis		Temperature	Temperature for the experimental conditions (°C)
	Concentration	The catalyst concentration along with its units of measurement	**Organisms**	Culture medium	The bacterial exposure medium
**Experimental conditions**	Temperature	Synthesis prevailing temperature. In case of a range of temperatures, the median value is reported (°C)		Species	The species exposed
	Order of reagent	The order of reagents in the synthesis process		Strand	The specific strand of the organism
	Conditions of light	Dark, light or sunlight exposure	**Outcome**	Bacteria reduction	The bacteria reduction along with its units of measurement (i.e., reduction in mm) and standard deviation
**External energy**	Energy	Addition of energy (through heating, sonication, ice bath, incubator, etc) or no addition of external energy		Bacterial methods	The methodology followed for the evaluation of the antibacterial efficiency
	Power	In case of microwave or sonication, the power used (W)	**Toxicological studies followed**		
	Stirring	In case of stirring applied further split into magnetic, mechanic or shaking, etc	**Exposure conditions**	Exposure dose	The exposure concentration along with its units of measurement (mg/ML)
	Stirring Speed	The rpm of stirring applied		Exposure duration	The exposure duration of the experiment (h)
	Pressure autoclave	The amount of pressure applied (psi)	**Cell culture information**	Cell line	Cell line name
	Ultrasound	In case of sonication, the frequency used (kHz)		Cell type	Cancer or normal and the specific type (i.e., glioma, macrophages, vascular, etc)
	Treatment	Any post treatment of AgNPs such as drying, washing, centrifugation, etc		Cell origin	The origin of the cell line (human, animal, plant).
**Post treatment**	Solution	The solution where the treatment took place		Cell organ	The organ which the cell line represents
	Methods	The methods applied for the specific treatment recorded above		Multiwell	The number of wells in the study
	Speed	In case of centrifugation applied, the rpm speed	**Outcome**	Toxicological assay	Assay used to assess the toxicological endpoint
	Duration	The duration of the speed method (min)		Toxicological endpoint	The endpoint measured by the assay
	Temperature	In case of drying process followed, the temperature applied (°C)		Toxicological results	The endpoint results are followed by the standard deviation and its units of measurements
	Duration	The duration of the drying method (min)	Information of the studies such as the author, title, year, DOI are recorded. An initial notebook of 6202 rows was created.		
	Other	Information such as the storage conditions			
	Yield	The yield of synthesis reaction ( %)			

### 3.2 Data harmonization and split

Data extraction was followed by data harmonization, where the features were unified to create a cohesive dataset. Compatibility and cohesion are essential for ML model development and to enable feature exploration for insights from multiple studies we harmonized all units measurements for example minutes converted to hours for synthesis time and exposure duration (h), millilitre for volume (ML), millimolar for concentration (mM), Celsius degree for temperature (°C), watt for external power (W), pound per square inch for pressure (psi), kilohertz for frequency (kHz), and milligram per millilitre for exposure dose (mg/ML), where feasible. In some cases units harmonization was not possible such as concentration expressed in either mass or volume metrics (i.e., nanoparticles count or NPs per milliliter (NPs/ML) where not possible to convert into a single metric.

Concerning the categorical features, we harmonized them in distinct classes. For example,

• the synthesis process was divided into biosynthesis, micosynthesis, green synthesis, or general wet chemical synthesis.
• the capping agent feature was first classified into aggregated classes, for example *algae extract* aggregated class contains species such as: *Padina sp. marine* alga extract, *Noctiluca scintillan*s, *Ecklonia cava,* etc, or the *fungal extract* aggregated class contains extract from *Aspergillus flavus*, *Emericella nidulans*, *Macrophomina phaseolina*, etc. In a second step, features were engineered to capture information on organic versus inorganic capping agent: organic contains information such as plant, fungal, fruit extracts, biopolymer or bacteria. The same approach was followed for the reducing agent.
• The order of reagents was converted into a codified feature where the silver precursor is denoted with A; reducing agent with B; capping agent with C; catalyst with D or other to simplify its representation. CADB for example would signify the following order of reagents: *capping;precursor;catalyst;reducing*. In that manner, the steps of synthesis can be represented by a single feature.
• The data was then split into synthesis dataset, pchem dataset, toxicological dataset and antimicrobial dataset for ML purposes.

#### 3.2.1 Synthesis & targeting core size

For the synthesis dataset, briefly, from the nearly 900 rows of information (from 219 studies), phytosynthesis was the most common synthesis process reported (57.7 % of rows, 119 studies) followed by wet chemistry (26 % of rows, 58 studies) and biosynthesis (12.7 % of rows, 28 studies). Missing values in the dataset containing all the synthesis information and pchem information (no toxicological or antibacterial information) (Supplementary material*, synthesis tab*) were common. Roughly 89 % of the rows had single-step synthesis process (185 studies). When external energy was applied, heating was one of the most common (41 %, 93 studies) followed by sonication (10 studies) and microwave assistance (5 studies). Regarding the temperature applied during synthesis, nearly half of the values fall within the range 24–64 °C with seven studies applying > 100 °C. Regarding the light conditions, 86 % of the rows contained no information (we hypothesised that no special light conditions were applied). 29 studies reported dark conditions. The exact order of reagents was missing in 35 studies. The duration of synthesis was absent nearly 18 % (42 studies) of the rows, while the scale of synthesis (ML), 30 %. Features that were missing more than 80 % contained the precursor purity information. However, precursors’ concentration (expressed in mM) had few missing values, same as capping agent compound (mostly plant or chemical agents) and reducing agents. On the other hand, capping agent and reducing agent concentrations (mg/ML) had nearly 40 % missing values. The pH during synthesis was missing > 70 % of the row cases. Regarding the pchem properties, dissolution was missing the majority of the time (> 90 %), shape nearly 30 %, crystal structure > 65 %, zeta potential and hydrodynamic size measurements > 70 %, while crystallite size, polydispersity index and surface area were almost always missing. Core size had the lowest count of missing values, nearly 25 % (6 studies out of 219 did not report core size). Since core size had the most data completeness and is one of the most crucial factors affecting biological interactions, antibacterial capabilities, and toxicity, was selected as model output to be predicted from synthesis parameters inputs.

##### 3.2.1.1 Targeting core size

Rows with no core size data were dropped, and when information on the standard deviation or size distributions on the core size was available (92 studies provided SD), those rows were triplicated containing the average, the minimum and maximum core size. This resulted in a new dataset (Supplementary material*, core size tab*) containing 2113 rows with 17 input features (7 numerical and 9 categorical) showed in Table 2. The stirring speed, capping and reducing agent concentrations features were excluded from the modelling part due to substantial amount of missing values.

**Table 2:** Input features used to predict core size along with the missing values presented in that dataset and a description of the features.

Group of features	Input features	Missing values	Description (min – max, mean values), categorical examples
The synthesis method	Synthesis process	0.0 %	Such as phytosynthesis (59 %), micosynthesis (4.8 %), wet chemical (24.5 %), biosynthesis (11 %), etc
	Steps involved	0.0 %	Single step synthesis (85 %), or multiple steps
	Synthesis duration	12.8 %	0.008 – 336, 17.5 (h)
	Scale	24.5 %	1 – 510, 87.2 (ML)
External energy	Energy	0.0 %	Such as heating (46 %), sonication (5.2 %), microwave (1.2 %), ice bath, UV treatment (1.8 %), cooling, none, etc
	Stirring	0.0 %	Such as mechanic (17.3 %), normal stirring (15.2 %), magnetic (13 %), shaking (12.9 %), yes (when no additional information was provided, 1.9 %), etc
	Stirring Speed	49.0 %	0 – 5000, 71 (rpm)
Experimental conditions	Temperature	1.3 %	-4 – 200, 48.4 (°C). In case of no information in the article, room temperature 25 °C was inserted
Precursor	Concentration	1.0 %	0.1 – 1000, 6 (mM)
Capping agent	Classification	0 %	Such as plant (55 %), chemical (14.5 %), biopolymer (8 %), fungal extract (5 %), biomolecule (4 %), animal derivative (1.8 %), algae extract, etc
	Class	0.0 %	Organic (92 %), inorganic or none
	Concentration	41.0 %	0.005 – 1000, 45.7 (mg/ML)
Reducing agent	Classification	5.3 %	Such as plant (56 %), chemical (16 %), biopolymer (5 %), biomolecule, animal derivative, fungi extract, etc
	Class	5.3 %	Organic (83 %), inorganic
	Concentration	35.9 %	0.0025 – 500, 16.1 (mg/ML)
Experimental conditions	Order of reagent	17.5 %	Codified feature such as AB (44 %), CADB, BA (20 %), etc, where, A: precursors addition, B: reducing agent addition, C: capping agent, D: catalysts, E: other step
Post treatment	Treatment	3.8 %	Such as washed (42 %), no post treatment (43 %), dried (2.2 %), centrifuged, pH adjusted, filtration, etc
Outcome to be predicted	Core size	0.0 %	0.1 – 334.8, 28.7 (nm)

In the next step, missing values were imputed via iterative ML based imputation. During each iteration, the imputation model uses the imputed values from previous iterations along with other available features to improve the accuracy of predictions. This process continues for a predefined number of iterations or until convergence is achieved. The dataset was randomly stratified split into ten training (1479 rows) and test sets (634 rows) for training and validation ML purposes. Models were validated via 10 fold corss validation approach which provides valuable insights into the predictive power of a model on unseen data from the training set.

#### 3.2.2 Toxicological

In case of the toxicological dataset, the creation of a dataset targeting a toxicological outcome with synthesis parameters as inputs was not feasible (Supplementary material, toxicological tab). The diversity of outcomes did not allow the selection of a single outcome of sufficient size and data completness. For example, various endpoints were measures such as cell viability, Inhibitory Concentration 50 % (IC50), Lethal concentration 50 % (LC50, LC90), minimum inhibitory concentration (MIC), lipid peroxidation, apoptosis, rate of haemolysis, scavenging activity, proliferation, etc. IC50 expressed as mg/ML, was the most commonly reported outcome in our case (20 studies), resulted only in nearly 500 rows of information. Thus, we did not proceed further with the toxicological case.

#### 3.2.3 Antibacterial efficiency

From the 219 studies, 134 of them provided an antibacterial efficiency assessment using diverse endpoints such as biofilm inhibition/formation (8 studies, endpoint expressed as % or CFU/ML), LC50 (2 studies), growth rate, inhibitory concentration (IC10 or IC50, 4 studies), minimum bactericidal concentration (MBC, 6 studies), minimum inhibitory concentration (MIC, MIC50, MIC90, 28 studies with endpoint expressed as mg/ML, % or NPs/ML, mM, etc), zone of inhibition (ZOI, 88 studies), etc. Based on the analysis, ZOI (mm) was selected as the endpoint to be modelled which represents a a circular area around the spot of the agent in which the bacteria colonies do not grow (expressed in mm) and indicates the effectiveness of the antimicrobial agent. The model captures the efficiency againste multiples strains providing a comprehensive understanding of its overall effectiveness and generalize findings. It may be more appropriate to develop separate predictive models for each bacterial strain for a more focused and accurate assessment of the relationship between antimicrobial agents and bacterial growth inhibition. However, due to limited availability of data for individual strains we combined information to assess the overall antimicrobial activity in a holistic persepctive.

Rows absent with ZOI values were dropped, and when information on the standard deviation on the ZOI results were available (15 studies provided SD), those rows where triplicated contain the average, the minimum and maximum ZOI values. This resulted in a new dataset (1251 rows with 25 features, 9 numerical and 16 categorical) shown in Table 3 (Supplementary material, antimicrobial tab). Scale synthesis, capping and reducing agent concentrations and stirring speed were excluded from the modelling part due to substantial amount of missing values.

**Table 3:** Input features used to predict zone of inhibition along with the missing values presented in that dataset and a description of the features.

**Group of features**	**Input features**	**Missing values**	**Description (min – max, mean values), categorical examples**
**The synthesis method**	Synthesis process	0.0 %	Green synthesis, phytochemical synthesis (70 %), wet chemical (9 %), biosynthesis (8.2 %), etc
	Steps involved	0.0 %	Single step synthesis (95 %), or multiple
	Synthesis duration	11.3 %	0.008 – 168, 16.2 (h)
	Scale	40.6 %	3 – 510, 107.2 (ML)
**External energy**	Energy	0.0 %	Such as heating (31 %), autoclave (4.2 %), sonication (1 %), microwave (3.7 %), ice bath, UV treatment
	Stirring	0.0 %	Such as mechanic (18.3 %), normal stirring (28.7 %), magnetic (10.7 %), shaking (5.2 %), etc
	Stirring Speed	49.0 %	0 – 5.000, 71 (rpm)
**Experimental conditions**	Temperature	3.8 %	20 – 200, 44 (°C) in case of no information in the article, room temperature was inserted
**Precursor**	Concentration	2.4 %	0.1 – 25,000, 19.2 (mM)
**Capping agent**	Classification	0.0 %	Such as plant (64 %), chemical (8.7 %), biopolymer, biomolecule, fungal extract (7.3 %), algae extract (7.8 %), etc
	Class	1.0 %	Organic, inorganic
	Concentration	34.1 %	0.02 – 500, 68.6 (mg/ML)
**Reducing agent**	Classification	1.4 %	Such as plant (65.7 %), chemical (9.5 %), biopolymer, biomolecule, animal derivative, algae extract (7.8 %), etc
	Class	1.4 %	Organic, inorganic
	Concentration	41.6 %	1.0 – 500, 36.4 (ML)
**Experimental conditions**	Order of reagent	7.1 %	Codified feature such as ABE, CADB, CAB, etc, where, A: precursors addition, B: reducing agent addition, C: capping agent, D: catalysts, E: other step
**Post treatment**	Treatment	0.2 %	Such as none, (51.6 %), washed (26.6 %), dried (4.2 %), centrifuged, pH adjusted, filtration (3.8 %), etc
**Pchem properties**	Shape	9.2 %	Such as spherical (76.8 %), prism, cubic (4.8 %), hexagonal, triangular, etc
	Core size	9.4 %	0.68 – 334.8, 32.1 (nm)
	Core size (method)	10.3 %	Such as TEM (51.3 %), SEM (22.8 %), AFM (6 %), FETEM (5.7 %), FESEM (2.2 %), etc
	UV-Vis peaks of NPs	6.1 %	280 – 780, 429 (nm)
**Exposure conditions**	Exposure dose	16.2 %	0.00001 – 1000, 9.2 (mg/ML)
	Exposure duration	13.8 %	3 – 168, 26.8 (h)
**Organisms**	Culture medium	17.3 %	Such as Corkborers, Luria Bertani, Agar, Mueller Hinton, etc
	Species	0.0 %	Such as *E. coli*, *Staphylococcus*, *Bacillus*, *Klebsiella*, *Salmonella*, *Candida*, etc
**Outcome to be predicted**	Bacteria reduction (endpoint: zone of inhibition)	0.0 %	0 – 80, 12.7 (mm)

### 3.3 Machine learning exploration

PyCaret regression library [70] was utilized to train various ML algorithms (linear regression, decision tree regression, random forest regression, and support vector regression, among others). Models parameterazation or optimization was not performed since the chosen algorithms have few hyperparameters and are relatively straightforward. In addition, the emphasis of the study is placed on the exploration of the feasibility of modelling approach using literature data rather than the parameter optimization. The models were trained using the training sets, which consisted of subsets of the available data (70 %). A cross-validation approach to evaluate the models’ robustness was used to validate models iteratively, with each fold serving as a validation unseen test set. This process was repeated ten times, ensuring that each fold was used also as the validation set. During cross-validation, various metrics to assess the accuracy and generalization capability of the models were assessed such as the root mean squared error (RMSE), mean absolute error (MAE), and R-squared (R^2^). The RMSE measures the average magnitude of the prediction errors and provides an indication of how well the model fits the data. The MAE represents the average absolute difference between the predicted and actual values and is useful for understanding the model’s overall accuracy. The R^2^ score quantifies the proportion of variance in the target variable that is explained by the model and indicates the goodness of fit.

#### 3.3.1 Core size prediction

The performance metrics of the 10-fold cross-validation results of the top five model’s out the 18 regression algorithms contained in the PyCaret library, shown in Table 4**.** The regression algorithms performed similarly with random forest and Extreme Gradient Boosting reaching during the 10-fold cross a R^2^ ≈ 0.70, RMSE≈ 16.85 and MAE≈ 9.10 followed by Extra Trees Regressor.

**Table 4:** Top five machine learning regression algorithms validation results metrics (mean values of 10-fold cross validation approach).

Model	Acronym	MAE	RMSE	R^2^
Random Forest Regressor	rf	9.11	16.82	0.70
Extreme Gradient Boosting	xgboost	9.06	16.89	0.70
Extra Trees Regressor	et	9.03	17.19	0.68
Decision Tree Regressor	dt	9.09	17.6	0.66
Light Gradient Boosting Machine	lightgbm	10.12	18.20	0.66

**Fig. 1:**
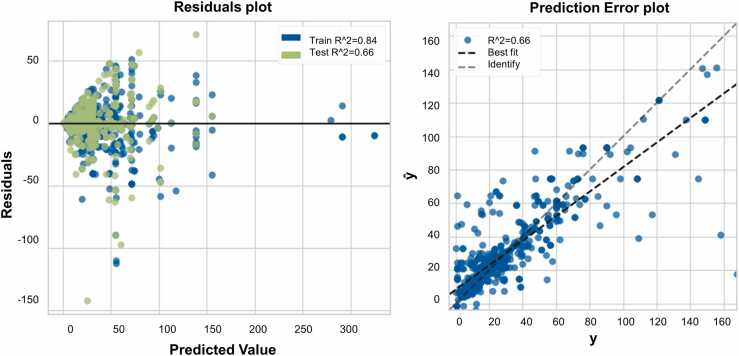
Residuals plot (left) and prediction errors plot (right) for random forest model. Train set: 1479 rows, test set: 634 rows. The figure represents one instance of the ten fold validation process.

Prediction error plot of random forest model (right) and residuals plot (left), of one individual instance of the ten subsets for visualazation purposes, are shown in Fig. 1. The blue dots on residuals plot represent the training set instances where the model is trained on 70 % of the data and shows an R^2^ value of 0.84. The green dots represent the test set instances where the model is tested on 20 % of the data as a test set and shows an R^2^ value of 0.66. The residuals plot allows to identify areas of the target that may be more or less prone to errors by displaying the difference between residuals on the y-axis and the dependent variable on the x-axis. The plot indicates a good-performing model since the residuals are randomly scattered. In the right panel, the actual targets from the dataset are plotted against expected values produced by random forest model in a prediction error plot. Comparing this plot to the 45 degree line (identity line), where the prediction exactly matches the model, allows diagnosing the regression model fit. The plot shows a good fit of the model with an R^2^ value of 0.66 displaying a correlation between actual core size values and its predicted values.

**Fig. 2:**
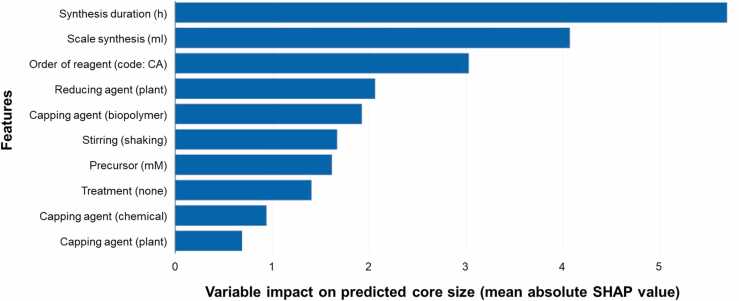
Top 10 variables impact on predicted core size values (mean absolute SHAP value).

We computed the mean absolute SHAP (SHapley Additive exPlanations) value as a measure to compare the influence of features in the model. The mean absolute SHAP value represents the average impact of a feature on the model’s predictions across all instances. By comparing the mean absolute SHAP values across different features, we gained insights into their relative importance in predicting the target variable. Upon analyzing the results, we observed that certain features exhibited higher mean absolute SHAP values, indicating their stronger influence on the model’s predictions (Fig. 2). These features collectively play a more significant role in determining the target variable and had a larger impact on the model’s output. Conversely, features with lower mean absolute SHAP values contributed less to the predictions and had a relatively smaller influence.

• The synthesis duration showed the highest SHAP value indicating its strong influence on the prediction values of the core size. The duration plays a crucial role in determining the structural and morphological characteristics of AgNPs; longer synthesis durations often result in larger particle sizes. The duration is closely linked to the kinetics and reaction pathways involved in the formation of NPs, due to a balance between growth and stabilization processes of NPs associated to reducing and capping agents [73]. Different synthesis durations can influence the rates of nucleation, growth, aggregation, or surface modification processes.
• The scale of synthesis followed second which could be explained by its function in determining the reagents interactions during synthesis. Changing the synthesis scale can affect the local ratios of reagents, which can influence the nucleation and growth kinetics, particle size distribution, and local composition [74]. It can also be attributed to its influence on the quantity- and composition-dependent NPs properties.
• The order of reagents was ranked third. The order can affect the nucleation and growth processes involved in synthesis [75]. Controlling the sequence of reagent addition, can influence the formation, capturing critical information about the kinetics and thermodynamics of nucleation and growth, and making it a significant predictor.
• The nature of the capping and reducing agent appeared high, since they provide information about AgNPs chemical nature that influences the surface chemistry and properties of the nanoparticles [76]. Consequently, the classification or the type of capping and reducing agent becomes a crucial predictor for modelling these nanoparticle characteristics, leading to high SHAP value. The reducing capping agent derived from plant is the most relevant in the top ten features, while the nature of the capping agent is followed in as biopolymer > chemical > plant.
• Stirring mode, concentration of precursor and type of post-synthesis treatment also appeared in the top ten SHAP values.

#### 3.3.2 Zone of inhibition prediction

The performance metrics results of the top five model’s of PyCaret's regression moduleshown in Table 5**.** The regression algorithms performed similarly during the 10-fold cross validation reaching a R^2^ ≈ 0.72, RMSE≈ 4 and MAE≈ 2.5 with Light Gradient Boosting Machine. Similar results were observed with random forest and extra tress regressor algorithm.

**Table 5:** Top five machine learning regression algorithms validation metrics (mean values) via 10-fold cross validation results.

Model	Acronym	MAE	RMSE	R^2^
Light Gradient Boosting Machine	lightgbm	2.49	4.07	0.72
Random Forest Regressor	rf	2.31	4.10	0.70
Extra Trees Regressor	et	2.27	4.20	0.68
Extreme Gradient Boosting	xgboost	2.35	4.432	0.65
K Neighbors Regressor	knn	3.02	4.71	0.61

**Fig. 3:**
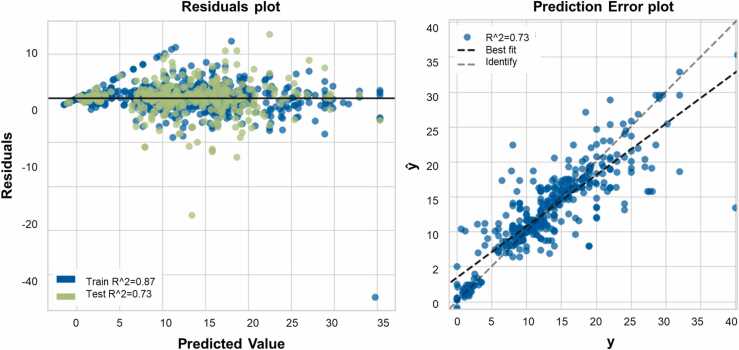
Residuals plot (left) and prediction errors plot (right) for lightgbm regression model. Train set: 875 rows, test set: 376 rows.

Prediction error plot (right) and of residuals error plot (left) of lightgbm regression model are shown in Fig. 3. The blue dots represent the training set instances where the model is trained on 70 % of the data and shows an R^2^ value of 0.87. The green dots represent the test set instances where the model is tested on 20 % of the data as a test set and shows an R^2^ value of 0.73. The plot shows fairly uniformly distributed residuals against the target in two dimensions indicating a good performing model. The prediction error plot shows a good fit of the model with an R^2^ value of 0.73 displaying a correlation between actual ZOI values and its predicted values.

**Fig. 4:**
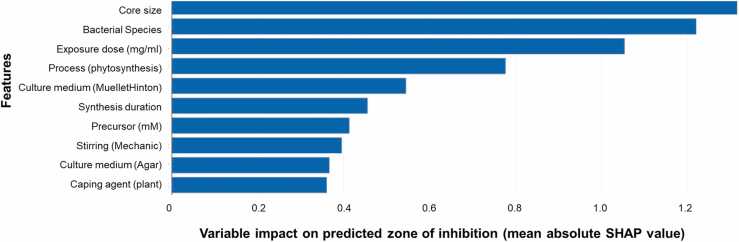
Variable impact on predicted bacterial reduction (mean absolute SHAP value).

We computed the mean absolute SHAP (SHapley Additive exPlanations) value as a measure to compare the influence of features in our model Fig. 4.

• Core size highest SHAP value in predicting the ZOI suggests its strong influence on the models’ predictions. The model reflected what is known by mechanistic investigations. Smaller NPs with larger surface areas have increased contact area with bacterial cells, enhancing the chances of interactions. The core size affects the diffusion and penetration of NPs into bacterial cells. Smaller NPs can more readily penetrate the bacterial membrane, being internalized, accessing intracellular targets, and disrupting essential processes, causing cell damage [77].
• The core size can influence the targeting and specificity of NPs towards bacteria, which can explain the bacterial species features appearing second. For instance, certain core sizes may exhibit stronger interactions with specific bacterial strains or surface receptors, leading to enhanced antibacterial activity [78], [79]. Understanding the size-dependent targeting capabilities is crucial for predicting the antibacterial efficiency of NPs.
• Exposure dose appeared third in the SHAP, since the antibacterial activity of NPs often exhibits a concentration-dependent behavior. Higher exposure concentrations provide a higher concentration of NPs (higher bioavailability), enabling more extensive interactions with bacterial cells and facilitating stronger inhibition of bacterial growth.
• The core size affects various properties of NPs, such as solubility, stability, and aggregation behavior. These properties can influence the dispersibility and behavior of NPs in the bacterial environment, impacting their antibacterial efficacy which can explain the type of culture medium appearing as top influential features (Muellet Hinton > Agar medium). For example, smaller NPs may exhibit higher stability and remain dispersed, ensuring sustained antibacterial activity [80], [81].
• Synthesis process and parameters also appear in the top five, directly linked to the final size (as mentioned in a previous session).

Consequently, the core size, bacterial species, culture medium and synthesis process become critical predictors for modelling the antibacterial efficiency, leading to their high SHAP values. Synthesis duration, stirring and capping agent also appeared in the top ten SHAP values.

## 4 Discussion

AgNPs are used in various products, such as antibacterial soaps, wound dressings, bandages, medical devices, certain cosmetics and personal care products (creams, lotions, deodorants, and shampoos) for their antimicrobial and antioxidant properties [82], [83], [84], [85]. Those functionalities have been attributed to their unique pchem properties, thus manipulating those or predicting the properties during the synthesis stage is stage is crucial for the final nano-enabled products. Overall, the ability to manipulate and/or predict pchem properties during the synthesis stage empowers researchers and manufacturers to create materials with tailored functionalities, optimized performance, and enhanced applicability in various industries. This approach also paves the way for innovative and advanced materials that can revolutionize technologies. By leveraging computational approaches, researcher can uncover synthesis parameters and pchem combinations that could enable the development of materials with tailored properties. In addition, predicting pchem properties during synthesis can lead to more efficient and cost-effective processes. By avoiding the need for trial and error in experimental synthesis, researchers can save time, resources, and materials. AgNPs can be synthesized through various methods and the synthesis process can be complex, involving multiple parameters that influence the resulting NPs characteristics. Addressing this challenge necessitates the development of efficient predictive models that can capture the relationship among all the synthesis variables. In this study, a first attempt of utilizing synthesis data manually extracted from the literature with ML approaches has been reported. The data-driven approach has benefits but also carries some limitations.

### 4.1 Benefits of a data driven approach

A modelling approach allows for the systematic investigation of AgNP synthesis by considering a wide range of parameters simultaneously. This holistic perspective facilitates a comprehensive understanding of the synthesis process, enabling the identification of optimal parameter combinations and conditions for desired AgNPs properties. ML, offers a valuable approach to be incorporated into the synthesis of AgNPs. It can effectively handle diverse datasets, capture nonlinear relationships, and make predictions [86], [87]. By utilizing ML algorithms, researchers can consider multiple synthesis parameters and their interactions, enabling accurate predictions of AgNP properties based on given synthesis parameters, helping to guide and prioritize experimental efforts [88]. It becomes possible to explore virtually the effects of different synthesis conditions combinations without conducting numerous time-consuming and costly experiments [89]. This speeds-up the synthesis process, reduces trial-and-error experimentation, and provides insights into the most influential parameters [90]. This approach promotes a more informed and data-driven approach to AgNP synthesis, leading to improved control over NPs characteristics and functionality. ML can aid in the discovery of novel AgNP formulations by guiding researchers towards unexplored regions of the parameter space [91]. This can lead to the identification of new NPs and functionalities that were not readily apparent through traditional synthesis approaches. The collaboration between traditional synthesis methods and ML-based approaches opens up new possibilities for innovation, efficiency, and impact in the field of materials science and nanotechnology. The feature importance analysis also provided valuable quantitative insights into the underlying factors that contribute significantly to the prediction of the outputs. By identifying them, deeper insights into the synthesis process and understand the parameters that have the strongest impact on the properties of AgNPs can be gained. This knowledge helps in streamlining the synthesis process, contributing to the development of more comprehensive and accurate models for predicting and optimizing AgNP synthesis. However, it is important to acknowledge limitations regarding data availability, generalizability, and assumptions made by the models.

### 4.2 Limitations of the data driven approach

The ML models developed in this study are trained and validated using a specific dataset collected from the literature. The applicability and generalizability of these models to other synthesis conditions, NPs systems, or antibacterial agents should be approached with caution. Therefore, further validation and testing on independent datasets are necessary to assess the model’s robustness and generalizability. The selection of features used in the models is critical for their predictive performance [92]. In this study, certain features were chosen based on their data completeness (missing values %). However, other important features may not be considered or available in the collected dataset. It is important to consider a broader range of features and explore their potential impact on the model’s performance in future studies such as the yield of reaction and other pchem properties or atomistic descriptors. While ML models can provide insights into feature importance and their impact on predictions (e.g., SHAP values), they do not inherently provide causality for example high SHAP values associated with certain features indicate their influence on the model’s predictions, but do not establish causal relationships. Therefore, caution should be exercised when interpreting the results and making causal claims based solely on the model outputs. For example, the synthesis duration is the most relevant feature to predict the final core size, followed by the synthesis scale and the order of reagents in the process [93], [94], [95]. Reducing and capping reagents' typology and concentration are less relevant in the final outcome compared to the three features mentioned above. Conducting experiments under controlled conditions using the predicted optimal synthesis parameters can help validate the model’s predictions and assess their practical utility. Addressing these limitations and further exploring the predictive performance of the models in real-world scenarios will be crucial for the practical application and wider acceptance of ML approaches in the field of NPs synthesis and antibacterial research.

### 4.3 The main limitation of data driven approaches: data

One of the main limitations of this study is the reliance on manually collected data from the literature. The availability and quality of the data might vary [96]. This lack of data poses challenges for researchers and hinders the development of accurate predictive models [97], [98]. It becomes difficult to establish general guidelines or best practices for achieving desired NPs characteristics. The accuracy and completeness of the data depend on the reporting and documentation of the synthesis procedures in the literature. Data harmonization is a crucial step in the development of ML models [99]. It involves standardizing and integrating diverse datasets from various sources to ensure compatibility and consistency, identifying common parameters, variables, and units of measurement across the studies. The lack of standardized reporting formats across different studies introduces variability in the collected data. Therefore, harmonizing the data is essential to create a unified and cohesive dataset for model development. It is crucial to collect comprehensive and diverse datasets that cover a wide range of synthesis conditions and nanoparticle properties to ensure the accuracy and generalizability of the models.

### 4.4 Future directions

To overcome the scarcity of data, researchers can adopt strategies to generate and share synthesis data. Collaboration among researchers and institutions can facilitate the pooling of data from different studies and contribute to a collective knowledge base [100]. Additionally, efforts to publish detailed synthesis protocols and characterization data can help bridge the data gap and foster reproducibility in the field. Furthermore, emerging techniques such as high-throughput experimentation and automated synthesis platforms can aid in generating large volumes of data on AgNPs synthesis. These approaches can rapidly explore a wide range of synthesis conditions and provide valuable insights into the effects of various parameters on nanoparticle properties. Sharing such data openly can encourage collaboration, accelerate progress, and enable the development of machine learning models and predictive tools [89]. To achieve harmonization, standardizing the data involves converting various units of measurement into a consistent format. For example, ensuring that reagent concentrations are expressed in the same units (e.g., molar concentration) or that the same approach is used for NPs characterization. This step minimizes discrepancies arising from different reporting conventions. Aligning the parameters involves mapping and matching the terminologies used in different studies. Synthesis parameters, such as reagent names, reducing agents, stabilizing agents, and experimental conditions, need to be harmonized to ensure consistency across the dataset. This may involve creating a standardized vocabulary or ontology to facilitate data integration and fairness. Strong efforts are ongoing in the field of nanosafety towards the principles of Findability, Accessibility, Interoperability and Reusability (FAIR) data and their importance for modelling studies and data-driven safe and sustainable application of nano- and advanced materials [100], [101], [102], [103].

Another emerging concept in the nanotechnology computational field is the use of read-across structure-activity relationship (RASAR) [69]. The integration of RASAR in the synthesis of AgNPs with ML models enhances the predictive capabilities and the efficiency of the models. RASAR leverages the similarity between chemical structures and their corresponding activities, enabling the extrapolation of properties to new or untested compounds. In this context, RASAR can be applied to complement the abovementioned models and enable the prediction the pchem properties, antibacterial efficiencies, and toxicological profiles of AgNPs beyond the specific data points manually extracted from literature sources. RASAR extends the predictive scope by considering structural similarities with known compounds, providing a broader understanding of the relationships between synthesis parameters and endpoints.

## 5 Conclusions

This study employed a data-driven approach to predict the properties of AgNPs and their antibacterial efficiencies from synthesis parameters. The models were trained and validated using manually collected data from the literature, and their performance was evaluated using various metrics. The feature importance analysis helped identify the influential variables in predicting the core size and zone of inhibition outputs. Factors such as synthesis duration, scale of synthesis, and the choice of capping agents were found to be important predictors in the models. The generalizability of the models to different datasets and experimental conditions should be further investigated. The development of standardized reporting formats for synthesis data would facilitate data collection and promote the reproducibility of studies in this field. Despite these limitations, the study showcases the potential of ML and their predictive capacity. The findings contribute to the growing body of knowledge in nanoscience and provide a foundation for further exploration and optimization of NPs synthesis processes by providing a data asset to be further explored and enriched. Ultimately, the integration of ML and experimental validation holds promise in advancing the development of materials, providing indication on how to implement a Safe-by-Design approach at the earliest stages of innovation.

## CRediT authorship contribution statement

Conceptualization, I.F., L.F., I.Z.and A.L.C.; methodology, I.F., L.F., I.Z., A.B. and M.V.; software, I.F.; validation, I.F., L.F., I.Z., A.B. and M.V.; formal analysis, I.F.; investigation, I.F., L.F., I.Z., A.B. and M.V.; resources, A.L.C.; data curation, L.F., I.Z., A.B. and M.V.; writing—original draft preparation, I.F., L.F., I.Z., A.B. and M.V.; writing—review and editing, I.F., L.F., I.Z., A.B., M.V. and A.L.C.; visualization, L.F.; supervision, A.L.C.; project administration, A.L.C.; funding acquisition, A.L.C. All authors have read and agreed to the published version of the manuscript.

## Declaration of Competing Interest

The authors declare that they have no known competing financial interests or personal relationships that could have appeared to influence the work reported in this paper.

## Footnotes

2 https://github.com/IrieIrie/Design-rules-AgNPs
